# COVID-19 self-testing in Brazil and the imminent risk of underreporting cases

**DOI:** 10.1590/0037-8682-00287-2023

**Published:** 2023-10-20

**Authors:** Laura Alves Ribeiro Oliveira, Miguel Junior Sordi Bortolini, Ernesto Akio Taketomi, Rafael de Oliveira Resende

**Affiliations:** 1 Universidade Federal de Uberlândia, Instituto de Ciências Biomédicas, Uberlândia, MG, Brasil. Universidade Federal de Uberlândia Instituto de Ciências Biomédicas Uberlândia MG Brasil; 2 Universidade Federal do Acre, Centro de Ciências da Saúde e do Desporto, Rio Branco, AC, Brasil. Universidade Federal do Acre Centro de Ciências da Saúde e do Desporto Rio Branco AC Brasil; 3 Fundação Oswaldo Cruz, Instituto Oswaldo Cruz, Rio de Janeiro, RJ, Brasil. Fundação Oswaldo Cruz Instituto Oswaldo Cruz Rio de Janeiro RJ Brasil; 4 Instituto Nacional de Ciência e Tecnologia em Neuroimunomodulação, Rio de Janeiro, RJ, Brasil. Instituto Nacional de Ciência e Tecnologia em Neuroimunomodulação Rio de Janeiro RJ Brasil

**FIGURE 1:**
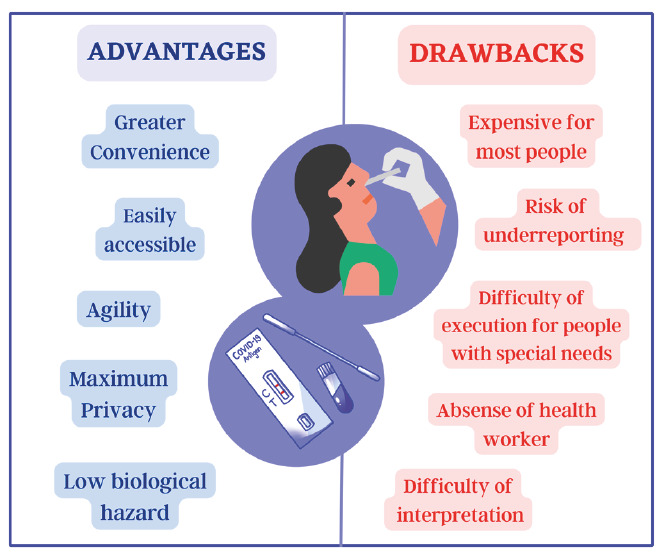
Main advantages and limitations of self-tests for COVID-19.

Dear Editor,

Since the onset of the coronavirus disease 2019 (COVID-19) pandemic, several strategies for identifying and isolating positive cases have been developed to control viral transmission. Among these strategies, immune-based assays, particularly point-of-care diagnostic kits, have assumed a pivotal role. Notably, self-administered tests have emerged as convenient alternatives, allowing users to conduct tests in their homes without visiting diagnostic laboratories or being assisted by healthcare practitioners^1^. The popularity of these tests relies on their ability to provide results within minutes, in contrast to other immunological tests or the gold standard reverse transcription-polymerase chain reaction test, which can take several hours or days^2^^-^^4^. However, this convenience is accompanied by a responsibility that rests solely with users who may encounter difficulties in execution or interpretation, particularly elderly individuals or those with cognitive limitations. Furthermore, as these tests are not funded by public health systems, their price can be prohibitive for people with low income or financial difficulties. Additional advantages and limitations/drawbacks of self-testing are shown in Figure 1.

To ensure the safety and effectiveness of self-tests designed for the detection of severe acute respiratory syndrome coronavirus 2 (SARS-CoV-2) antigens in Brazil, the National Health Surveillance Agency (Anvisa) has implemented a regulatory framework that encompasses registration procedures, commercialization protocols, distribution standards, and usage guidelines for self-tests. Since January 2022, Anvisa has endorsed the market entry of 62 self-test kits, thereby significantly contributing to the diagnostic chain of public health management^5^. Among them, a notable portion of 59 self-tests rely on nasal swabs for antigen detection, whereas a smaller subset of three employ saliva as the biological sample^6^. Typically, these kits comprise a membrane-based immunological cassette, buffer solution, and a swab or collector, accompanied by user-friendly instructional manuals. The interpretation of the results is based on the presence of a test line in conjunction with a control line on the cassette display. As required by Anvisa’s directives, the sale of self-test kits is limited to pharmacies and health markets, as they undergo regular inspections by the agency^6^. Moreover, the acquisition process for these kits is simple, eliminating the need for appointments or medical prescriptions. 

The inclusion of self-testing as an additional tool to conventional serological diagnostic methods has gained recognition as a valuable strategy for managing local outbreaks because it enables the identification of viral circulation within specific groups in the community, thereby facilitating the prompt implementation of isolation measures^6^. In addition, as vaccination campaigns progress globally, including Brazil, COVID-19 self-testing remains pivotal in identifying positive cases among vaccinated and unvaccinated populations. However, a major concern regarding the commercialization of these kits is the potential lack of self-reporting by users, who may choose not to seek a confirmatory diagnosis in the case of a positive result. This negligence bias poses a challenge in detecting cases and subsequently controlling SARS-CoV-2 outbreaks^7^. Within a global state, despite the limitations of the test, individuals have attained a clear understanding of the significance of self-testing^8^^,^^9^. Most European populations reported a willingness to undergo testing once a week^10^. Nevertheless, comprehensive measures have been established to assist in the voluntary disclosure of positive cases via apps or online platforms, supported by educational campaigns that underscore the importance of such reporting of COVID-19 epidemiological concerns. In Brazil, the majority of citizens in São Paulo are also engaged in self-testing and would be able to notify the corresponding health unit in case of a positive result, although 10% of the respondents expressed reluctance to disclose^11^. However, dedicated platforms for autonomous case reporting are not available, highlighting the potential requirement for guidelines to encourage immediate reporting of positive results following test completion. 

The symbiotic arrangement provided by public-private partnerships has played a substantial role in addressing the challenges posed by the COVID-19 pandemic. A holistic approach to mitigating the underreporting bias associated with positive results provided by self-testing involves the implementation of effective public health measures, such as including explicit statements regarding the benefits of reporting outcomes in instruction manuals. Additionally, supportive initiatives directed towards app developers, fiscal incentives for general companies, and convenience to their employees, such as paid leave during quarantine or other related benefits should be implemented. Cashback refunds or loyalty programs for customers; continued education programs; and awareness campaigns on social media, television, radio, and other media providers, in association with leaders and public figures as role models, would also be valuable. 

In summary, ensuring that users are informed about the importance of notifying health authorities is indispensable for taking appropriate measures for disease control and monitoring. Although the World Health Organization has stated the end of the global health emergency and the COVID-19 scenario in Brazil remains unclear, many self-test kits may emerge. As the availability of these kits continues to expand, it is crucial to implement transparent measures to raise awareness among the population regarding the importance of reporting a positive outcome. This finding supports the adoption of public policies to control disease incidence.
